# Morphological Features of the Anther Development in Tomato Plants with Non-Specific Male Sterility

**DOI:** 10.3390/biology9020032

**Published:** 2020-02-17

**Authors:** Inna A. Chaban, Neonila V. Kononenko, Alexander A. Gulevich, Liliya R. Bogoutdinova, Marat R. Khaliluev, Ekaterina N. Baranova

**Affiliations:** 1 All-Russia Research Institute of Agricultural Biotechnology, Timiryazevskaya 42, 127550 Moscow, Russia; inna_chaban@rambler.ru (I.A.C.); nilava@mail.ru (N.V.K.); a_gulevich@mail.ru (A.A.G.); bogoutdinova_lr@rambler.ru (L.R.B.); 2 Moscow Timiryazev Agricultural Academy, Agronomy and Biotechnology Faculty, Russian State Agrarian University, Timiryazevskaya 49, 127550 Moscow, Russia

**Keywords:** *Solanum lycopersicum* L., microspore development, male gametophyte, pollen grain ultrastructure, pseudo-pollen, phenotypical flower anomalies

## Abstract

The study was devoted to morphological and cytoembryological analysis of disorders in the anther and pollen development of transgenic tomato plants with a normal and abnormal phenotype, which is characterized by the impaired development of generative organs. Various abnormalities in the structural organization of anthers and microspores were revealed. Such abnormalities in microspores lead to the blocking of asymmetric cell division and, accordingly, the male gametophyte formation. Some of the non-degenerated microspores accumulate a large number of storage inclusions, forming sterile mononuclear pseudo-pollen, which is similar in size and appearance to fertile pollen grain (looks like pollen grain). It was discussed that the growth of tapetal cells in abnormal anthers by increasing the size and ploidy level of nuclei contributes to this process. It has been shown that in transgenic plants with a normal phenotype, individual disturbances are also observed in the development of both male and female gametophytes. The reason for the developmental arrest of some ovules was the death of endosperm at different stages of the globular embryo. At the same time, noticeable hypertrophy of endothelial cells performing a secretory function was observed. In the ovules of transgenic plants with abnormalities, the endothelium forms a pseudo-embryo instead of the embryo sac, stimulating the development of parthenocarpic fruits. The data obtained in this study can be useful for a better understanding of the genetic and molecular mechanisms of cytoplasmic male sterility and parthenocarpic fruit development in tomatoes.

## 1. Introduction

Pollen development is one of the most important processes in the life cycle of plants, since only normally formed pollen contributes to proper fertilization and the formation of full-fledged seeds and fruits. However, under adverse conditions or any effects on plants, violations primarily occur in the anther development, which leads to the formation of sterile pollen.

Most studies focus on the genetic aspects of cytoplasmic male sterility (CMS). As is known, the process of pollen formation involves several stages and is regulated by a vast number of genes [1,2,3,4,5]. Numerous genes and proteins associated with the abortion of microspores and pollen [6,7] and the molecular mechanisms underlying various male sterile phenotypes have been characterized [6,8,9,10,11,12]. However, the morphological and cytological aspects of pollen sterility have not been investigated enough, although quite a lot of research has been devoted to the cause of pollen sterility and the reasons for its manifestation in different plants [13].

Previously, we performed a morphological, anatomical, and cytological study of anomalies in the flowers of transgenic tomatoes, which sometimes arise as a result of the genetic transformation procedure [14,15,16,17]. It was shown that the reason for the appearance of most anomalies in the flowers of transgenic tomatoes is a violation of the predetermined generative meristem growth. This leads to various kinds of disturbances in the formation of both gynoecium and androecium. It was found that pollen in most of the studied transgenic plants were sterile due to the arrest of asymmetric mitosis in microspores. Nevertheless, apparently, due to the hormonal deviations in cells around the embryo sac, so-called pseudo-embryonic tissue is formed in the unfertilized ovules from the inner epidermis of the integument. In turn, it stimulates the ovary to develop and form parthenocarpic fruits [18].

The aim of this work was to identify the cytoembryological features of anthers and pollen development to establish morphological factors for pollen sterility at key stages of the androecium and gynoecium development in transgenic tomato plants with anomalies of generative organs, as well as in transgenic tomatoes without visible phenotypic anomalies, but with partially sterile pollen. These model tomato plants were previously used to study pseudo-embryo formation in the ovules of parthenocarpic fruits [18].

## 2. Results

**Figure 1:**
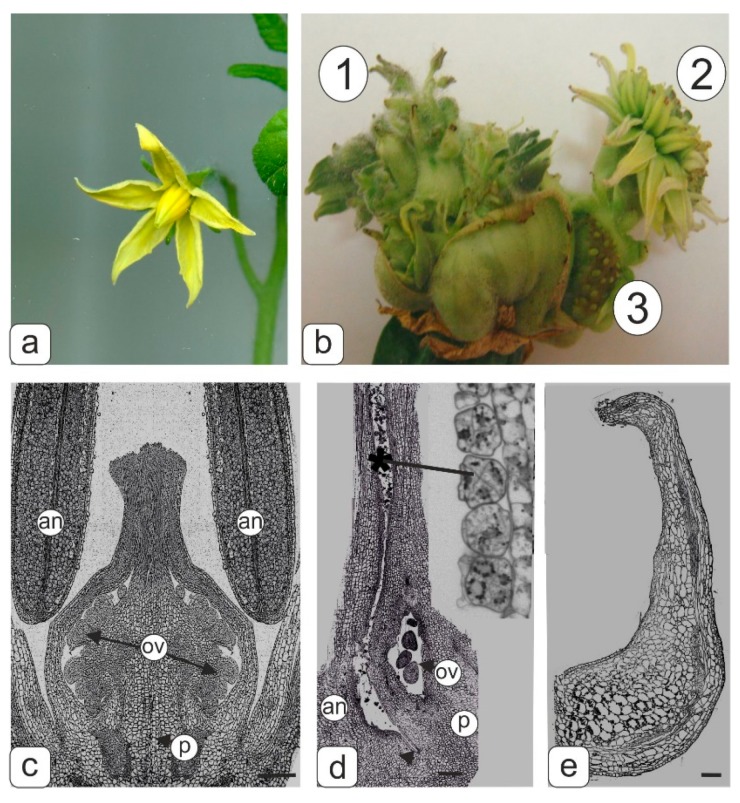
Flower phenotype of control and transgenic tomato plants and semi-thin longitudinal sections of control and abnormal transgenic tomato flowers fragments at the microsporogenesis initial stage. (**a**) Flower of control tomato line. (**b**) Flowers with abnormal morphology of transgenic tomato lines formed from abnormal fruit; secondary flower formed at the base of the primary ovary in the transgenic tomato line: 1–upper small flower buds at abnormal multitier tomato fruit; 2–flowers assembled in pseudo inflorescences; 3–sterile seed buds. (**c**) Middle section of pistil and stamens in control tomato line. (**d**) Accreted pistil and stamen from pseudo inflorescences. Abnormal microspore tetrads (asterisk) are visible inside the pollen sac of the anther. (**e**) Abnormal stamen. Symbols: an–anther; ov–ovules, p–pistil. Bar—500 μm (**c**); 200 μm (**d**,**e**).

The anthers of the flowers from the control tomato plants could be clearly observed to form microsporocytes before meiosis (Figure 1a,c). In the flowers of transgenic tomato plants with abnormalities, the pestle was noticeably larger compared to the control. This is due to the fact that additional generative shoots sprout through the ovary from the receptacle. As a result of this, the fruit forming from the ovary burst, and its small ovules appeared on the surface (Figure 1b (3)). In addition, new generative shoots with separate small flowers formed on randomly growing shoots (Figure 1b (1)). Some shoots combined into a kind of multi-flower inflorescence resembling chrysanthemum-like flowers (Figure 1b (2)).

An accretion of stamens with a pistil was observed in flowers of a compact “inflorescence” of the “chrysanthemum” type (Figure 1b (2)). Microsporocytes appeared to be forming in pollen sacs of such anther, however, obviously, the formation of tetrads failed during meiosis, because abnormal degraded microspores were present in tetrads inside the pollen sac (Figure 1d). The extreme extent of abnormality in the androecium formation was manifested in the complete reduction of anther pollen sacs (Figure 1e).

**Figure 2:**
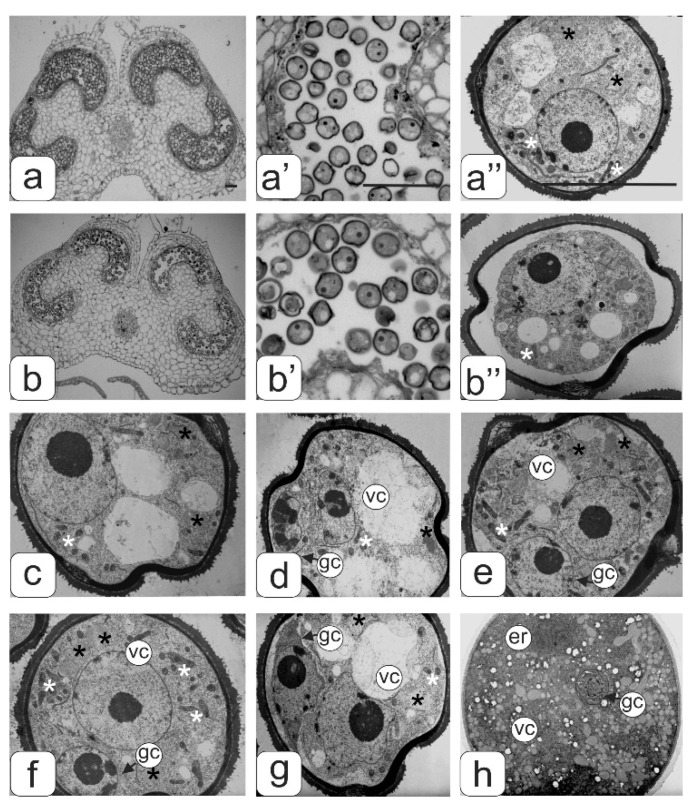
Semi-thin cross sections of anther from the control (**а**,**a’**,**a’’**) and transgenic tomato plants without abnormalities (**b**,**b**’,**b**’’) at the microspore stage. Microspore ultrastructure at some asymmetric mitosis stages (**с**–**g**), and mature pollen grain ultrastructure (**h**). Symbols: er–endoplasmic reticulum; gс–generative cell; vc–vegetative cell; black asterisks–plastids, white asterisks–mitochondria. Bar—100 μm (for semi-thin sections: a–b’) and 10 μm (for ultrathin sections: a”; b”; c–h).

The disturbances in the androecium development in the flowers of transgenic tomatoes with phenotypic abnormalities most often occurred after normally passing meiosis when the microspores converted into pollen grains. This was manifested in a large number of deformed microspores and abnormal pollen in anthers, as well as in the formation of anthers themselves (Figure 2).

Figure 2 shows transverse sections of anthers and pollen from tomato flowers of the control line (Figure 2a) and a transgenic line with a normal phenotype (Figure 2b) at the asymmetric mitosis stage. This asymmetric mitosis was observed in almost all of the microspores in the anthers of the control flowers, while not all microspores passed to the next stage of development in the anthers of transgenic plants without abnormalities. A gradual compression of the protoplast characteristic for plasmolysis was observed in some of them. Additionally, such underdeveloped microspores later on degraded (Figure 2b’b’’).

**Figure 3:**
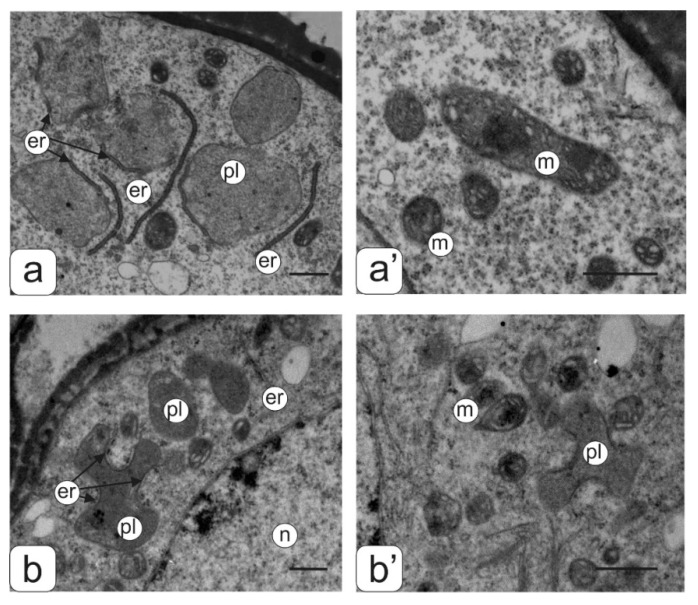
Ultrastructure of the cytoplasmic fragment from the microspore of normal (**a**,**a**’) and transgenic (**b**,**b**’) tomato anthers with an abnormal phenotype. Plastids membranes–ER association (**a**,**b**) and mitochondrial ultrastructure (**a’**,**b’**). Symbols: er—endoplasmic reticulum, m—mitochondria, n—nucleus; pl—plastids. Bar—1 μm.

Ultrastructural analysis of pollen (Figure 2c–h) in some key phases of asymmetric mitosis did not reveal significant changes in the structure of the cytoplasm, with the exception of some individual details. The microspore cytoplasm was quite dense at all stages of asymmetric mitosis, with a uniform arrangement of ribosomes. The plastid numbers were relatively low; they had a diverse shape—round or slightly amoeboid. The plastid stroma had a moderate density and practically did not contain lamellae. In some plastids small plastoglobules inclusions were found (Figure 3a). In the early stages of mitosis many plastids are in tight contact with single endoplasmic reticulum (ER) cisternae forming peculiar complexes. ER cisternae were separated from plastids and arranged separately from each other in the form of single and rather short cisternae during mitosis. Mitochondria in sections had an elongated or round shape. They contained a dense matrix and numerous expanded cristae (Figure 3a’). Large vacuoles prevailed.

**Figure 4:**
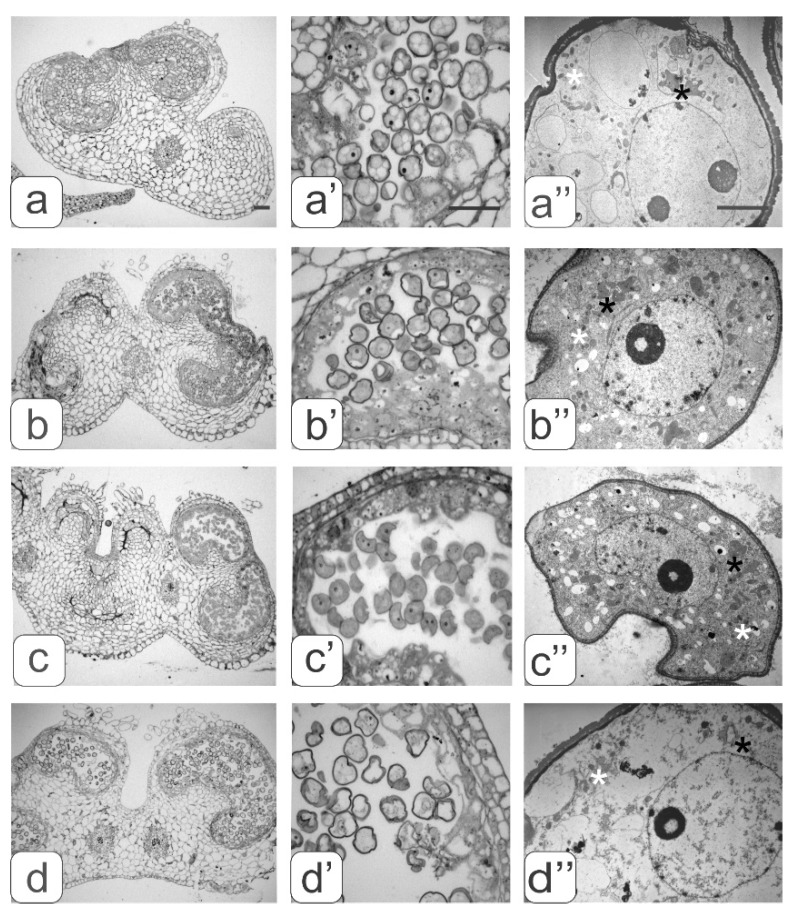
Cross-sections of anthers with various types of developmental abnormalities (**a**–**d**). Pollen sacs (**а**’–**d**’) and microspore ultrastructure (**a’**’–**d’’**) from these anthers. black asterisks—plastids, white asterisks—mitochondria. Bar—30 μm (for semi-thin sections: a–d’) and 3 μm (for ultrathin sections: a”–d”).

Different variants of developmental anthers in the flowers of transgenic tomatoes with abnormalities and the microspores in them are presented in Figure 4.

Abnormalities in anther development manifested in different ways. We identified four types of abnormalities:

1. underdevelopment or complete atrophy of one or more pollen sacs (Figure 4a);
2. merging of two pollen sacs into one common sac and degradation of the other two (Figure 4b);
3. coalescence of two stamens along the entire length and atrophy of pollen sacs in these merged anthers (Figure 4c);
4. integration of two stamens into one with complete atrophy of two pollen sacs from each of them as evidenced by two vascular bundles (Figure 4d).

An analysis of these data suggested that various disturbances in anther formation affected the degree and type of anomalies in the structure of the developing microspores.

The main evidence of anther ontogenesis violation from the transgenic plants with an abnormal phenotype was the fact that almost all microspores stopped their development at this stage. An asymmetric mitosis was not observed in any of the microspores. In addition, many microspores acquired a curved shape, as well as various defects in their internal structure.

A strong vacuolization of the cytoplasm and rarely distributed ribosomes were observed in the microspores from the anther of the first type. Large vacuoles significantly reduced the volume of the cytoplasm (Figure 4a”).

An abundant arrangement of ribosomes and a large number of small vacuoles, many of which contain osmiophilic inclusions, were viewed in microspores from the second type of anthers. (Figure 4b’’). Plastids are mostly amoeboid in shape. They have small plastoglobule inclusions and a dense matrix without lamellar structures (Figure 3b”). Many plastids were in contact with ER cisternae. Numerous mitochondria have a rounded shape, a dense matrix and elongated cristae (Figure 3b”).

Most microspores from anthers of the third type have a deformed (abnormal) shape, and their internal structure is very similar to the structure of the microspore cytoplasm from anthers of the second type, namely: a high density of ribosomes, a large mitochondrial number as well as small vacuoles in the cytoplasm (Figure 4c” and Figure 3b”). A significant part of the microspores had an abnormal shape in anthers of the fourth type. They contained large vacuoles and a partially degraded cytoplasm. Such microspores were apparently in the process of degradation (Figure 4d”).

**Figure 5:**
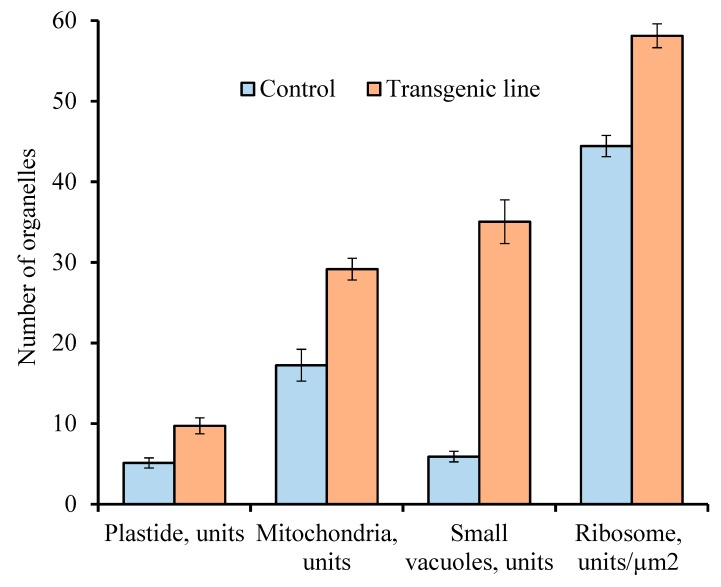
Comparative analyses of the number for different organelles per microspore section of the control plants and transgenic tomato line with abnormalities. Data points are the means (*n* ≥ 150) and their standard errors (α = 0.05).

A comparative morphological analysis revealed significant differences in cytoplasm structure between intact microspores from anthers of the second and third variants and developing pollen from anthers of the control tomatoes (Figure 4). These differences were expressed both in the structural organization of various organelles and in their number per cell. The average number of ribosomes, plastids, mitochondria, and small vacuoles in the cytoplasm of microspores per section was estimated (Figure 5).

It was found that the number of aforementioned organelles was significantly higher in the microspores from transgenic abnormal plants (with violations of type 2 and 3) than in the cytoplasm of microspores from the control plants.

Along with microspores in all anther types, the degree of tapetum development was analyzed. In the anthers of the control (Figure 2a’) and transgenic tomato plants with a normal phenotype (Figure 2b’), in the phase of asymmetric microspore division, the tapetum was observed at the final development stage during degradation. Tapetum cells did not degenerate in all types of abnormal anthers from the transgenic tomato plants (Figure 4c’–f’). On the contrary, one can observe an increase in their number and size. In this case, asymmetric nuclear division in microspores does not occur.

**Figure 6:**
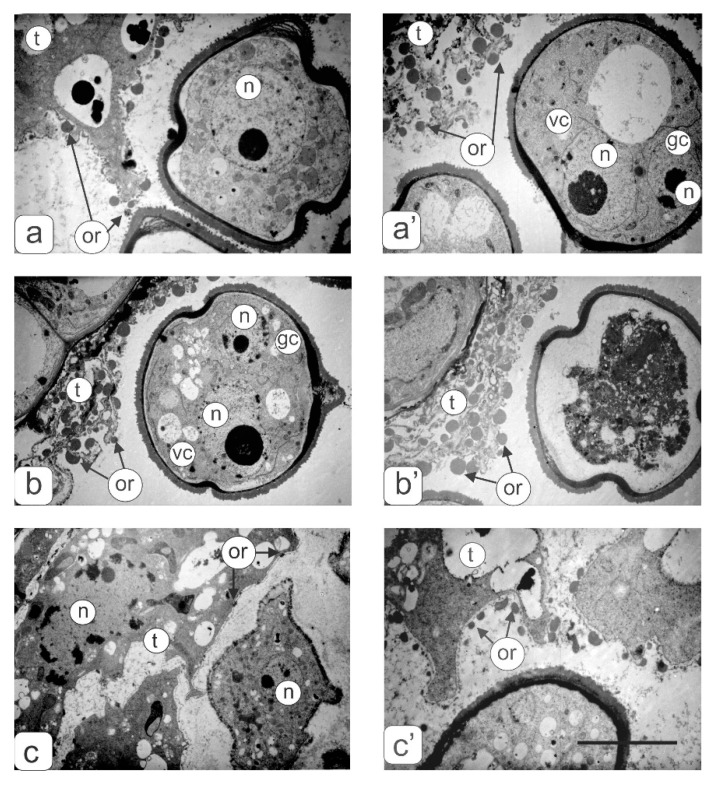
Ultrastructural fragments of anthers transverse sections in the tapetum zone: (**a**,**a’**) control tomato plants at the microspore (a) and asymmetric mitosis (a’) stages; (**b**,**b’**) transgenic tomato plants with a normal phenotype at the mitosis stage; (**c**,**c’**)—transgenic tomatoes with three (c) and two (c’) types of anther abnormalities. Symbols: t—tapetum; gс—generative cell; vc—vegetative cell; n—nucleus; or—orbicules. Bar—6 μm.

For a more detailed identification of the tapetum cells structure and nearby pollen, an electron microscopic study of this zone from anthers of the control as well as transgenic tomato lines with two and three types of abnormalities were carried out (Figure 6). The membrane fragments and numerous orbicules were preserved from degraded tapetal cells in the anthers of the control (Figure 6a’) and transgenic tomato plants without phenotypic anomalies (Figure 6b,b’). Tapetal cells from the anthers of transgenic tomato plants with anomalies remained intact (Figure 6c,c’). However, there was a significant difference in the number and size of the orbicules between two and three types of disturbances in transgenic tomato plants.

**Figure 7:**
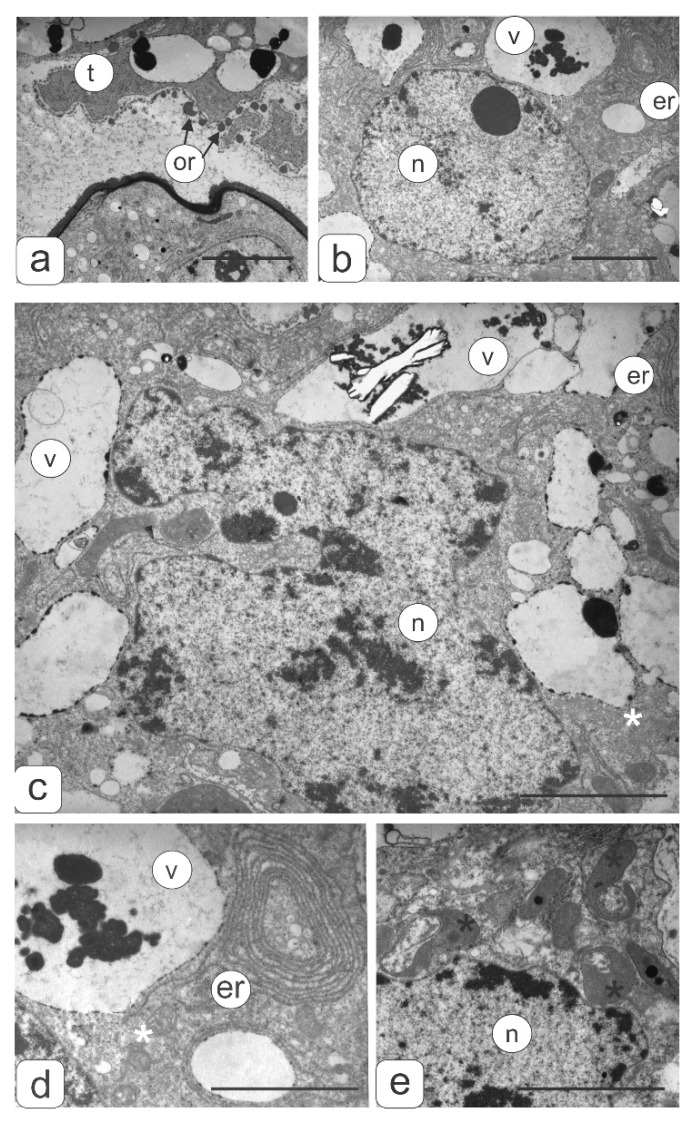
Tapetal cell ultrastructure of transgenic tomato plants (2 disturbances types) with violations of flower morphology. (**a**) Fragment from contact zone of tapetal cell and microspore. Orbicules are visible on the tapetum surface. (**b**) Fragment of tapetal cell. (**c**) Polyploid nucleus of tapetal cell. Tapetal cell fragments with ER (**d**) and plastids (**e**). Symbols: er—endoplasmic reticulum; v—vacuole; n—nucleus; or—orbicules; black asterisks—plastids, white asterisks—mitochondria. Bar—2 μm.

Figure 7 shows the tapetal cells ultrastructure in anthers from two disturbance types of transgenic tomato plants. Tapetal cells contained large polyploid nuclei (Figure 7b,c) and a dense cytoplasm with a large number of organelles and numerous vacuoles (Figure 7a–e). Plastids showed the most diverse form, and had a dense matrix, rare lamellae and plastoglobule inclusions (Figure 7e). Mitochondria also had a diverse shape and well-developed cristae (Figure 7c,d). Dictiosomes were accompanied by a large number of vesicules around them against the background of a dense matrix (Figure 7c). Long ER cisternae penetrated the entire cytoplasm and often formed ring-shaped complexes (Figure 7d) mainly around the vacuoles. In vacuoles, large osmiophilic inclusions, crystals, and also deposits on the inner surface of the tonoplast were observed.

**Figure 8:**
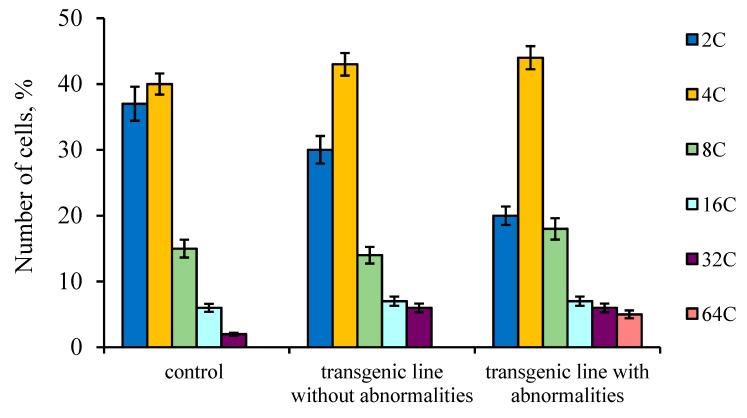
DNA content of isolated tapetal cells nuclei from control tomato anthers as well as transgenic tomato line without and with abnormalities. Data points are the means and their standard errors (α = 0.05).

The cytophotometry method was used to measure the DNA content in the nuclei of tapetum cells from the anthers of the studied tomato plants to determine the ploidy level (Figure 8). The data showed that the largest number of highly polyploid tapetal cells (32C–64C) was observed in the anthers of transgenic tomato lines with phenotypic abnormalities.

**Figure 9:**
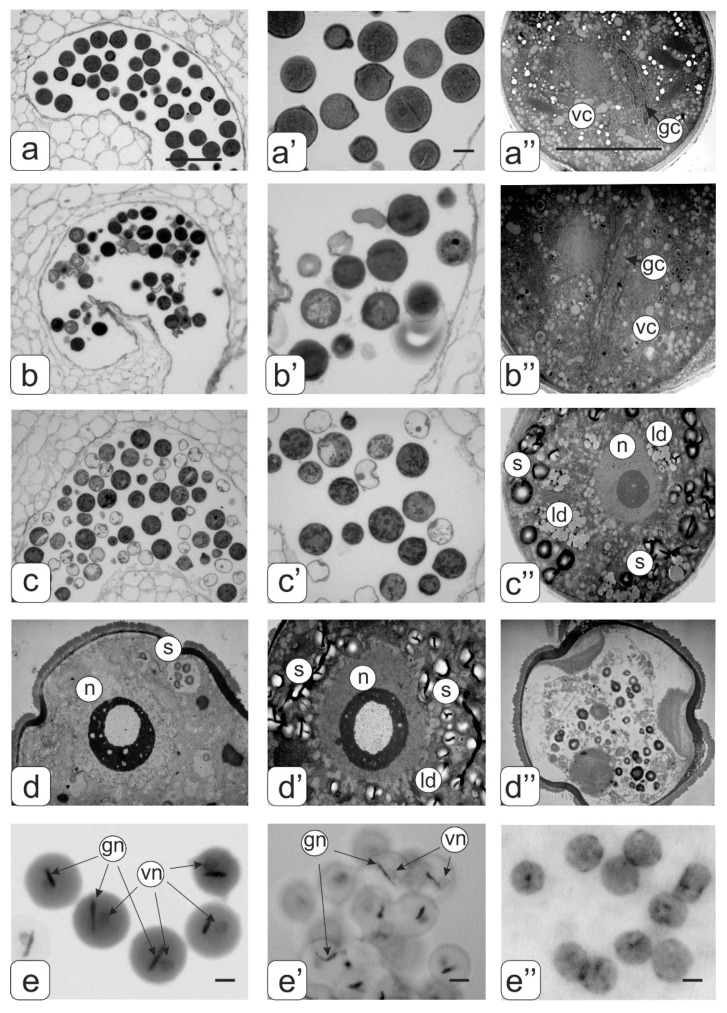
Cross-sections of mature anthers and ultrastructure of pollen in tomato flowers. Pollen sacs (**а**) and pollen grain (**a’**,**a”**) from control tomato plants; pollen sacs (**b**) and pollen grain (**b’**,**b”**) from transgenic tomato line with normal phenotype; pollen sacs (**c**) and hypertrophic microspore (**c’**,**c”**) from transgenic tomato line with abnormalities. Ultrastructure of different abnormal microspores in transgenic tomato plants (**d**,**d’**,**d”**). Pollen grains of control (**e**) as well as transgenic tomato plants with normal (**e’**) abnormal phenotypes (**e’’**) stained by the Feulgen. Symbols: gс—generative cell; vc—cell; n—nucleus; gn–generative nucleus; vn—vegetative nucleus; ld—lipid drops, s—starch. Bar: a, b, c—25 μm; other—4 μm.

On the cross-sections of the anthers, it can be seen that the amount of mature pollen grains with a normal structure in the transgenic lines was very different from the control tomato plants (Figure 9). Almost all pollen grains in the control tomato plants achieved full maturity (Figure 9а,a’). The cytoplasm was dehydrated in the mature pollen grains, and therefore cellular organelles were poorly identified. The dense cytoplasm contained amyloplasts with small starch grains, mitochondria and lipid drops. The ER was packed in stacks consisting of 15–17 parallel cisternae. The nucleus of the vegetative cell had a lobed shape, and was usually located in the center of the pollen grain. The generative cell had a fusiform generative cell and was located near the nucleus of the vegetative cell (Figure 9a”). This can be seen in the pollen preparations stained by the Feulgen (Figure 9e).

In the transgenic tomato plants with a normal phenotype, abortive pollen were present among ripened pollen grains (Figure 9b,b’). In general, mature pollen grain were similar to control tomato plants, but there were some differences in their structure, particularly in the ER structure and with fewer ER cisternae. The heterogeneity of pollen grains was visible on preparations stained by the Feulgen (Figure 9e’).

Among a large number of abortive pollen, there were pollens that were similar in appearance to mature pollen grains (looks like pollen grain) in the anthers of transgenic tomato plants with abnormalities (Figure 9c,c’). However, such pollen was in fact a hypertrophic microspore as shown by ultrastructural analysis (Figure 9c”). Such a microspore contains one large nucleus with a nucleolus; the cytoplasm is filled with large starch grains and aggregated lipid drops. Various variants of abnormal microspores are presented in Figure 9d–d’’. A clear localization of DNA was not detected by Feulgen staining (Figure 9e”).

**Figure 10:**
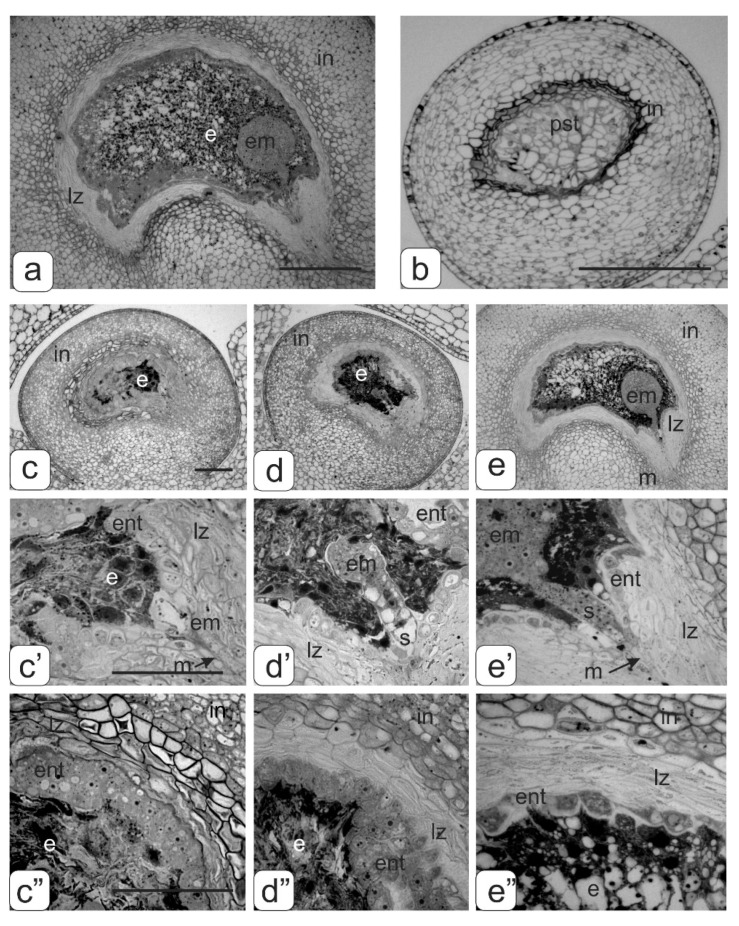
Disturbances of ovule development in transgenic tomato plants. (**a**)—globular stage development of the embryo in the control tomato plant; (**b**)—ovule with a pseudo-embryo in parthenocarpic fruits of transgenic tomato plants with abnormalities; proembryo stage (**c**,**c’**), stage of the early globular embryo (**d**,**d’**) and stage of late globular embryo (**e**,**e’**) in transgenic tomato plants without phenotypic anomalies; structure endothelium tissue on stage of the early embryo (**c”**,**d”**) and stage of late globular embryo (**e”**). Bar: a–e—100 μm; c, c’–e, e’—50 μm.

In the control tomato plants, the fruits with viable seeds were formed. The globular phase of seed development is shown in Figure 10a. Despite defective pollen, the transgenic tomato plants with abnormalities formed parthenocarpic fruit, which stopped in the development ovules. Such ovules instead of the embryo and endosperm contained pseudoembryogenic tissue formed by the endothelium (Figure 10b).

The fruits of the transgenic tomatoes without phenotypic anomalies, along with full-fledged seeds, also stopped in the development ovules of different sizes. When conducting embryological analysis of such ovules, it was revealed that the reason for stopping their development was premature degradation of endosperm cells in the different globular stage developments of the embryo: proembryo stage (Figure 10c,c’); stage of the early spherical embryo (Figure 10d,d’); stage of late spherical embryo (Figure 10e,e’). In addition, an increased number of endothelial cells were observed in the first two early stages (Figure 10c”,d”). At the stage of the late globular embryo, we could see the beginning of endothelium degradation (Figure 10e”).

**Figure 11:**
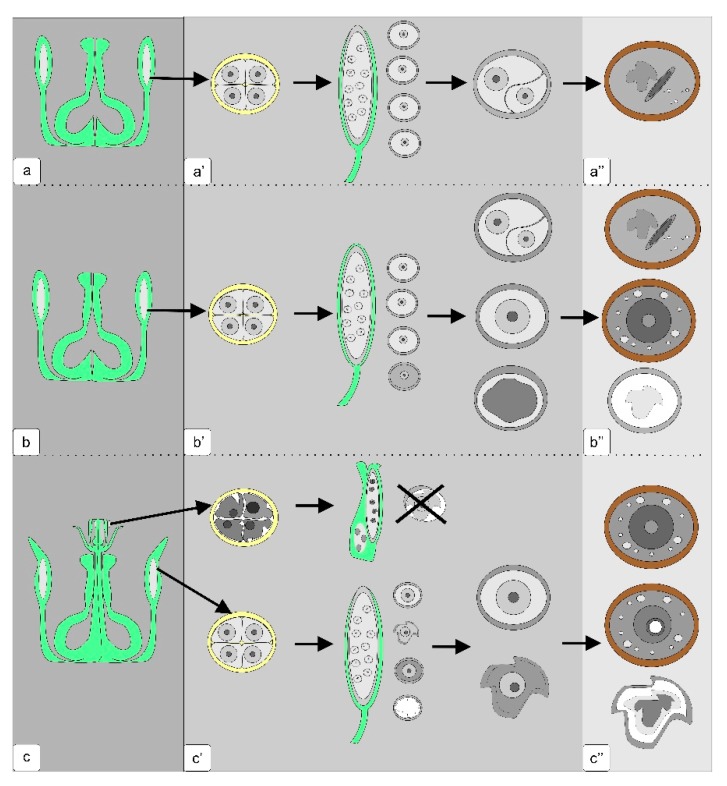
Microsporogenesis pattern in anthers of normal tomato plants (**a**,**a’**,**a’’**), transgenic plants without phenotypic abnormalities (**b**,**b’**,**b’’**) and transgenic plants with abnormalities in the generative organs (**c**,**c’**,**c’’**). The dark gray panel is a scheme of the generative organs of the flower; gray panel—the development of microspores from the exit from tetrad to the stage of asymmetric mitosis; light gray panel—the stage of mature pollen.

A generalized diagram of the pollen formation in anthers from the control and transgenic tomato flowers is presented in Figure 11.

## 3. Discussion

The use of light and transmission electron microscopy for the comparative structural organization of anthers, developing microspores and pollen in flowers from the control tomato line, transgenic tomatoes without noticeable phenotypic disorders as well as transgenic lines with abnormalities was carried out. It is known that the degree of anthers abnormalities from sterile plants depends on the stage at which the normal pollen development is interrupted [19]. In the present work, we analyzed microsporogenesis stages at which the main disturbances of the anthers and pollen development in the flowers of transgenic tomato lines were manifested. The study showed that in most flowers of all transgenic tomato lines meiosis in microsporocytes occurs normally. Disturbances in pollen development are already observed after the release of microspores from the tetrad. Only in some flowers of transgenic tomato plants with hypertrophically growing ectopic shoots were the abnormalities detected in the earlier stages of microsporogenesis (during the formation of sporogenous tissue or meiocytes in early meiosis). According to some research, disorders in plants such as cytoplasmic male sterility (CMS) are quite common [4,20,21,22]. At the same time, pollen sterility is often manifested at later stages of microsporogenesis (during the formation of the male gametophyte) [23,24]. A detailed anatomical analysis of the anthers structure in the flowers from transgenic plants with abnormalities revealed various defects in their development. Atrophy of one or several pollen sacs or their fusion was observed. In such various anthers, microspores formed with varying structural abnormalities. However, regardless of the microspore abnormalities, there was no subsequent asymmetric nuclear division with the formation of vegetative and generative cells. The cytoplasm of abnormal microspores contains significantly more plastids, mitochondria, ribosomes, and small vacuoles compared to the microspores of control tomato plants. It is believed that an increase in the number of mitochondria in a vegetative cell is one of the prerequisites for the maturation of normal pollen grains [25,26]. Based on this, it can be assumed that an increase in the number of mitochondria and other organelles in an abnormal microspore with blocked mitosis contributes to complete microspore development. According to some studies, mitochondria are cytoplasmic determinants of CMS in some plants. Changes in the interaction between the mitochondria and nucleus can significantly affect morphological changes in the cell during differentiation [5]. In abnormal microspores, asymmetric nuclear division and cytoplasmic redistribution does not occur. They accumulate a large number of storage inclusions. Such microspores reach the same size as normal pollen grains in most cases.

When studying the anthers of transgenic tomatoes without phenotypic abnormalities, fertile pollen grains and microspores showed signs of degradation, and microspores with asymmetric nuclear division, but forming pseudo-pollen, were revealed.

The tapetum plays important roles in anther and pollen development [13,20,27]. The tapetal cells during microsporocyte meiosis produce various enzymes and other compounds (lipids, sugars, pollen wall materials) essential to pollen formation [28]. Thus, abnormal tapetum development at early stages of microsporogenesis lead to dysfunctional mitosis [4]. It is also known that the tapetum developmental stage in normal anthers, as a rule, exactly corresponds to the stages of microsporogenesis. Any disturbances of this synchronous process can lead to premature degeneration and lysis of tapetum and, accordingly, to pollen sterility [13]. Normally, tapetum degrades in the late stages of microsporogenesis. It is believed that the tapetum degradation occurs by programmed cell death [29,30,31].

Many genetic studies address the causes of sterile pollen. Most authors indicate that predominantly abnormalities in the tapetum development and disturbances of its secretory function are the main factors that trigger CMS [4,13,27,32]. This is manifested in premature degeneration of the tapetum, or most often in long-term preservation or strong growth after microspore development. Many CMS plants are characterized by hypertrophied cells of the tapetal layer. For example, inhibition of tapetum degeneration in mutant rice anthers leads to impaired pollen membrane formation and subsequent degeneration of microspores [30,33,34]. Our comparative study showed that in anthers of the transgenic tomato plant with abnormalities, in which the microspore development stopped due to blocking cell division, the number and size of tapetal cells increased. In addition, the nuclear size of these cells was significantly larger than that of the control, which was confirmed by the results of cytophotometric cellular DNA-content measurements. In our opinion, an increase in the ploidy level of nuclei in tapetal cells may indicate the enhanced functional activity of tapetum and the delay in its degradation. Apparently, an increase in the functional activity of tapetal cells is associated with the continued development of microspores. In our opinion, tapetum hypertrophy and activation of metabolic processes contributes to further mononuclear microspore development, but not their degradation. The absence of asymmetric nuclear division in microspores occurs earlier than the manifestation of any structural changes in the tapetal cells.

Subsequently, we performed an anatomical analysis of immature seeds in the fruits of transgenic tomato plants without phenotypic anomalies, since along with fertile pollen grains, anthers also contain abnormal pollen without the male gametophyte, and are arrested in the development of microspores. The ovule stops its development in such fruits due to the death of endosperm cells. This mainly occurs in the early and late globular stage of embryonic development. These ontogenetic stages are the most critical in the ovule developments of most angiosperms and highly dependent on external environmental factors [35]. It can be assumed that the reason for arresting the ovule development in transgenic plants may be defects in the pollen structure or, most likely, disturbances in ontogenetic regulation of some anthers and ovules. Similar changes in ovule developments also occur during intraspecific and interspecific crosses of tomato [36] and other plants [16,17,37,38].

In our study, the role of endothelium in immature ovules is not fully understood. At the stages of the early embryo, the cells of the single-layer endothelium surrounding the dying endosperm look intact and in some places are located in two layers. This may indicate an increase in the functional activity of tapetal cells. In this connection, an analogy arises with tapetum hypertrophy in the abnormal anthers of transgenic tomatoes, in which a pseudo-pollen with its single nucleus is formed. The origin of these tissues varies markedly, but there is some functional similarity. Both anthers and ovules in the early developmental stages regulated secretion. The endothelium in the tomato ovule is the internal epidermis of integument, transformed into an independent tissue. In the early stages of embryo development, the endothelium separates and protects the developing endosperm from dying integumental parenchyma cells due to PCD [18]. Tapetal cells in anthers during pollen formation completely degenerates, while the endothelium in tomato ovules remains practically until the seed fully matures. Thus, tapetum as a secretory tissue exists until envelopes are formed in pollen. The endothelium also functioned only for a short time period as secretory tissue during the globular stage of embryogenesis.

A cuticle forms on the surface of the endosperm when embryo organogenesis occurs at the stage of laying down and development of the cotyledons. At the same time, the structural organization and functional specialization (from secretory to meristematic type) of endothelial cells also changes markedly. Most likely, with cell death in the endosperm in the late globular phase of development, endothelial cells do not change, but, apparently, they also begin to degenerate. From this we can conclude that the hypertrophy of tapetal cells and endothelium can only be during their functional activity. Therefore, with any disturbance of the anther and ovule development at these stages, a structural reorganization occurs in the cells of both tissues to enhance their metabolic activity. In the ovules of transgenic tomatoes with abnormalities, fertilization does not occur due to pollen sterility. However, such sterile pollen is a trigger for endothelial cell proliferation, which forms pseudo-embryonic tissue instead of the embryo sac, ovule growth as well as parthenocarpic fruit formation [15,39].

## 4. Materials and Methods

### 4.1. Plant Material

The tomato plant was from the *Solanum lycopersicum* L. line YaLF (control) and transgenic tomato plants expressing the *ac* gene, encoding chitin-binding protein were from *Amaranthus caudatus* L. with normal and abnormal phenotype, which was characterized by impaired development of generative organs. Putative transgenic tomato plants were produced by *Agrobacterium*-mediated transformation using pBI121ac binary vector [11]. The transgenic tomato lines with abnormalities were propagated by grafting lateral shoots formed on adult plants. Control and transgenic tomato plants were grown in plastic pots (5 L) filled with soil, at standard conditions (22–25 °C day-time temperature and 18–19 °C night-time temperature, humidity 60–70%, illumination 2500 lx). All vegetative generations were obtained in the same way.

### 4.2. Light Microscopy and Transmission Electron Microscopy

Ovaries and ovules at different stages of development were fixed for 1 day in 2.5% glutaraldehyde (Merck, Darmstadt, Germany) prepared on 0.1 M Sorensen’s phosphate buffer (pH 7.2) with 1.5% sucrose. Then samples were post-fixed in 1% OsO_4_ (Sigma-Aldrich, St. Louis, MO, USA), dehydrated in ethanol of increasing concentrations (30 > 50 > 70 > 96 > 100%), propylene oxide and embedded in a mixture of Epon-812 and Araldite (Merck, Darmstadt, Germany), by standard protocol. For light microscopy, semi-thin sections (1–2 µm) were prepared using glass knives and an ultramicrotome LKB-V (LKB, Sweden), placed on glass slides, stained with 0.1% methylene blue (Merck, Darmstadt, Germany), and embedded in epoxide resin. Samples were analyzed and photographed using an Olympus BX51 microscope (Olympus, Shinjuku, Tokyo, Japan) supplied with a Color View II camera (Soft Imaging System, Münster, Germany). For electron microscopy, samples were sectioned with a diamond knife using an ultramicrotome LKB-V (LKB, Sweden), placed on formvar-coated grids, and stained with uranyl acetate and lead citrate. Then ultrathin sections were analyzed and photographed by an electron microscope H-500 (Hitachi, Ibaraki, Japan).

### 4.3. Cytophotometry and Statistical Analysis

For evaluation of the ploidy level of pseudo-embryo cells, the ovules from green parthenocarpic fruits were isolated, incised with a razor blade, and fixed in ethanol/acetic acid mixture (3:1) for 3 h. Then, the ovules were treated with 5N HCl for 40 min at 22 °C and stained with Schiff reagent (Merck, Darmstadt, Germany), according to the standard procedure. Stained cells of the pseudo-embryo were visualized under the microscope, separated from other tissues, and used for evaluation of the DNA content using a SMP-20 cytophotometer Opton (Carl Zeiss, Obercochen, Germany) and 0.08–2.5 mm probes, and registered as arbitrary units. Three samples containing more than 200 nuclei were examined; each sample was prepared from four to five ovules. The program Statistica 5.0 (Round Rock, Texas, US), Student’s parametric criteria, and standard Microsoft Excel software were used for date evaluation.

## 5. Conclusions

The study revealed different types of anthers morphological abnormalities in the development of genetically modified (transgenic) tomato plants. It was shown that in most cases the pollen sterility cause was blocking the asymmetric mitosis in microspores. However, many of these single-core microspores will continue their differentiation through the reserve substances accumulation, forming pseudo-pollen. It was assumed that this process correlated with an increase in cell size and nuclear ploidy in the abnormal anthers tapetum.

We hope that the data obtained in this work may be useful in studies of the genetic and molecular foundations of CMS and parthenocarpy fruit development.
